# Supplementary material for: Deep microbial colonization during impact-generated hydrothermal circulation at the Lappajärvi impact structure, Finland
Source: Nat Commun. 2025 Sep 17;16:8270. doi: 10.1038/s41467-025-63603-y (PMC12443983; doi:10.1038/s41467-025-63603-y)
Supplement: Supplementary file 1 — Supplementary Information [file 41467_2025_63603_MOESM1_ESM.pdf]

Supplementary Information
To

Deep microbial colonization during impact-generated hydrothermal circulation at the Lappajärvi impact structure, Finland

Jacob Gustafsson[a*], Gordon R. Osinski[b], Nick M.W. Roberts[c], Jay Quade[d], Zhennan Wang[d] Martin Whitehouse[e], Heejin Jeon[e], Andreas Karlsson[e], Satu Hietala[f] and Henrik Drake[a*]

[a]Department of Biology and Environmental Science, Linnaeus university, 39182 Kalmar, Sweden, jacob.gustafsson@lnu.se; henrik.drake@lnu.se
[b]Department of Earth Sciences, University of Western Ontario, London, ON N6A 5B7, Canada,
[c]Geochronology and Tracers Facility, British Geological Survey, Nottingham, NG12 5GG, UK,
[d]Department of Geosciences University of Arizona, AZ 85721 Tucson, USA,
[e]Department of Geosciences, Swedish Museum of Natural History, 114 18 Stockholm, Sweden,
[f]Geological Survey of Finland, P.O. Box 1237, FI-70211 Kuopio, Finland,

Supplementary Text 1. Extended discussion of paleodepths for groundwater to reach 47.0±7.1 °C

Estimations of the paleogroundwater temperatures at the time of mineral precipitation are needed to attest that the determined formation temperatures (47.0 ± 7.1 °C) represent a cooling hydrothermal system. First we need to establish the paleodepth of mineral formation = 325 m, which is given by an estimated erosion maximum of 190 m (Hall et al., 2021) and the 135 m depth (calculated from drill core depth: 137 m and dip: 80°) of the sample with T = 47.0 ± 7.1 °C. Current groundwater temperatures at similar depths in the Fennoscandian shield bedrock, such as at Olkiluoto, Finland are ~10-11 °C and the geothermal gradient ~14 °C km^{-1} (Sedighi et al., 2014; Rosberg et al., 2021). Given this gradient, and applying an average near-surface groundwater temperature of 5 °C (Leppäharju, 2008), a depth of ~3.0 ± 0.5 km is needed to reach 47.0 ± 7.1 °C. As groundwater temperatures show climate-related variability (Mastrocicco et al., 2018), it is relevant to consider that in the late Cretaceous: (1) global air temperatures were higher than the current, by ~3 °C (Royer et al., 2004); and (2) that the Fennoscandian shield was at a latitude similar to the present-day Mediterranean (Merdith et al., 2021), a climate region with present-day groundwater temperatures of ~16 °C, with a couple of degrees local variation (Mastrocicco et al., 2018). Assigning increased paleogroundwater temperatures based on the latter assumptions would still need a depth of ~2.0 ± 0.5 km to reach 47.0 ± 7.1 °C. This is significantly deeper than the estimated depth of calcite formation and supports that calcite formation was related to a cooling impact-generated hydrothermal system.

Supplementary Text 2. Extended discussion on mechanisms that could explain the longevity of the heat source or pockets within the crater.

As was previously mentioned (see Introduction), various factors contribute to the longevity IGH systems within impact craters. There are three main potential heat sources that drive IGH systems (Kirsimäe and Osinski 2013; Osinski et al, 2013):

(1) Impact melt rocks and impact-melt bearing breccias. An enormous amount of energy is released during an impact event. Melting of large volumes of target rock occurs upon decompression from high shock pressures and temperatures (Grieve et al., 1977). Emplacement of impact melt can form up to 1-2 km thick superheated "melt sheets" with initial temperatures of >2370°C (Timms et al., 2017) occupying the annular "trough" surrounding the central uplift and form local thermal anomalies (Grieve et al., 1977).

(2) *Elevated geothermal gradients in central uplifts.* In complex craters (>3 – 4 km diameter on Earth), an elevated local geothermal gradient occurs due to the uplift of the target rock in the central uplifts (Kirsimäe & Osinski, 2013).

(3) *Energy deposited in the central uplift due to the passage of the shock wave.* As discussed by Kirsimäe and Osinski (2013), this potential source of heat for

hydrothermal systems is poorly constrained but is generally assumed to be minor relative to the other two heat sources.

The results of our study support an earlier study (Schmieder and Jourdan, 2013) that the hydrothermal system at Lappajärvi was exceptionally long-lived given the size of the structure. For comparison, the duration of the impact-generated hydrothermal systems at the similarly sized Haughton (23 km diameter) and Ries (24 km diameter) impact structures were ~50,000 (Trowbridge et al., 2024) and 250,000 years (Arp et al., 2013), respectively.

Schmieder and Jourdan (2013) suggested a number of variable factors that may have delayed the Lappajärvi crater cooling, including: (1) an elevated geothermal gradient due to impact-induced uplift; (2) residual heat generated by shock and friction within the central uplifted domain; (3) a potentially relatively "dry" state of the crystalline target rock, which lead to a relatively slow conductive heat transfer from the center of the structure; (4) crystallization heat released during hydrothermal alteration, (5) inherently low permeability of the target rock; and (6) the insulating effect of K-feldspar-hosting granite clasts, which are embedded within or located beneath a thick (≥145 m; Vaarma and Pipping, 1997) and potentially dense and impermeable impact melt sheet.

The two most recent estimates for stratigraphic uplift (SU) in complex impact structures from Kenkmann (2021) ($SU = 0.069D^{0.96}$) and Osinski et al. (2022) ($SU = 0.0849D^{0.7611}$), yields ~1.46 km and 0.93 km, respectively. Using an average geothermal gradient of 25 °C per km of depth, this results in temperatures of well under 50 °C for any scenario. Furthermore, given the similar size of the Lappajärvi, Haughton, and Ries impact structures, we suggest that the elevated geothermal gradient due to impact-induced uplift cannot account for the long duration of cooling at Lappajärvi. It is also unlikely that the residual heat generated by shock and friction within the central uplift would be that different at Lappajärvi (cf., Kirsimäe and Osinski, 2013).

This discussion leads us to conclude that the heat source for the Lappajärvi hydrothermal system was impact melt rocks and impact-melt bearing breccias, which has also been suggested earlier for the Haughton, Ries, and other medium-size structures (Osinski et al., 2013). What can explain the longevity of the hydrothermal system at Lappajärvi, or at least pockets of prolonged fluid flow, compared to the similarly sized Haughton and Ries structures? The simplest explanation is the difference in target rocks.

The Haughton and Ries impact structures formed targets with thick (~1.8 km and 0.8 km, respectively) sections of sedimentary rocks overlying metamorphic crystalline basement; whereas Lappajärvi formed in predominantly crystalline metamorphic rocks. This led to the generation of a thick (currently 145 m but originally likely much thicker) layer of silicate impact melt rock along with large amounts of impact melt-bearing breccia that filled the interior of the crater (see Fig. 1 in main manuscript). While the sedimentary rocks at the Haughton and Ries structures did undergo melting (Graup, 1999; Osinski and Spray, 2001), these products were dispersed to form particulate melt-bearing impactites. As a result, no coherent bodies of silicate melt rock are known at Haughton and only very small (a

few m in size) examples formed at the Ries. Furthermore, there is no evidence to date that impact melts generated from sedimentary rocks can reach the extreme temperatures (>2370 °C; Timms et al., 2017) of melt generated from crystalline rocks.

The properties of the target rocks at Lappajärvi may have also resulted in other factors that extended the duration of the hydrothermal system. Schmieder and Jourdan (2013) discussed how the potentially relatively "dry" state of the crystalline target rock promoted relatively slow conductive heat transfer from the central uplifted part of the impact structure, which would render Lappajärvi a relatively slowly cooled impact structure. In water-bearing targets, the latent heat of vaporization can enhance convective fluid circulation which effectively cools down the surrounding rock (Jõeleht et al., 2005; Kirsimäe and Osinski, 2013). Furthermore, although local deposition of phyllosilicate minerals and pyrite (Schmieder and Jourdan, 2013; Lehtinen, 1976; Naumov, 2005, present study) indicates an impact-induced hydrothermal system was developed at Lappajärvi, alteration was probably not uniformly distributed throughout the crater, which is evident from the fresh state of impact melt rock and at some depths also the impact melt-bearing breccias (cf., the Haughton structure; Osinski et al., 2005). This implies that hydrothermal circulation was localized at Lappajärvi, and convective heat transfer may not have been widespread in the crater.

Schmieder and Jourdan (2013) also discussed permeability of the target rock and the crater fill impactites, as numerical modeling demonstrate that low permeability values of the target rock prolong the theoretical duration of hydrothermal activity (Abramov and Kring, 2004, 2007; Osinski et al., 2013). Additionally, low permeability of hot rock and/or melt and leads slow heat removal by conductive heat (Kirsimäe and Osinski 2012). At Lappajärvi, the impact melt rock exhibits relatively low porosity, hydraulic conductivity, and fracture frequency, along with higher bulk density and P-wave velocity than the underlying impact melt-bearing- and lithic impact breccias (Kukkonen et al., 1992). In addition, the impact melt rock has a generally fresh condition, which could imply that the melt sheet had a low permeability shortly after the impact (Schmieder and Jourdan, 2013). The latter is further supported by the "layered" structure of the impact melt rock, with a ~90 m thick, almost vug- and vein free, intermediate impact melt rock layer with a glassy (perlitic) matrix (Kukkonen et al., 1992), which testifies to rapid quenching. The latter layer is bracketed by ~20-30 m thick top- and bottom-layers, which contain a high proportion of vugs and veins. In the present study, the oldest isotopic evidence for microbial activity was found in the bottom-layer of the impact melt rock, which contain abundant veins filled with secondary minerals, thus hinting a spatial association of the longevity of the hydrothermal system and the bottom layers, which remains to be explored. Ranges of $^{40}Ar/^{39}Ar$ ages recorded in single craters, have been interpreted to reflect differential cooling rates of different domains of the crater, such as impact melt bodies or sheets, a central uplift, an annular crater moat, or the rim region in complex impact craters (Kenny et al., 2019). For instance, the $^{40}Ar/^{39}Ar$ age spread of cooling Lappajärvi melt rocks of at least 1.1 ± 0.5 Ma (Schmieder and Jourdan, 2013), has been interpreted to record progressive cooling of different domains of the impact structure (Kenny et al., 2019).

Insulation of K-feldspar-hosting granite clasts enclosed within or capped underneath the thick, potentially dense and impermeable melt sheet, is another potential factor which could have caused slower cooling of the impact structure (Schmieder and Jourdan, 2013). However, since highly thermally conductive quartz is a major mineral constituent within granite, and quartz-rich clasts are present in the melt rock, the delaying cooling effect of the insulation of K-feldspar-hosting granite, we therefore considered minor.

Supplementary Text 3. X-ray diffraction method

The sample material was handpicked from the vugs (clay mineral sample) and hand-drilled material from a vein (calcite/siderite sample) and was then ground to powder in an agate mortar and was placed on a background free sample holder made out of silicon metal. X-ray powder diffraction data were recorded in a PANalytical X'Pert^3 Powder diffractometer system (Cu $K_{\alpha1}$-radiation) operated at 40 mA and 45 kV. The data was collected between 5 to 70 2θ° for 60 minutes with an X'Celerator strip detector. Background, peak searching and search & match-operations were performed using PANalytical HighScorePlus (4.7), peak positions were corrected against external Si (metal) standard (NBS640b). Mineral phases were matched against known reference patterns of minerals from COD (Crystallography Open Database, Gražulis et al., (2009)), and shown in Supplementary Fig. 7, and Supplementary Data 8.

Supplementary Text 4. Gas-chromatography / Mass-spectrometry

The calcite samples showing proposed microbial $\delta^{13}C$ composition was analyzed for preserved organic remains at the University of Göttingen, following protocol outlined in Reinhardt et al (2024), but since all drill core samples were found to be too contaminated to allow interpretation of ancient biosignatures, we have not included them in the main text or supplementary information.

Supplementary Text 5: U-Pb Geochronology

In situ U-Pb geochronology of individual calcite crystals was conducted over three analytical sessions at the Geochronology and Tracers Facility, British Geological Survey, following the method documented previously (Roberts and Walker, 2016; Roberts et al., 2017; Drake et al., 2017; Rochelle-Bates et al., 2021). The full data are provided in Supplementary Data 6 and the analytical conditions are provided in Supplementary Data 7. Spots were placed using zonation visible in Back-Scattered-Electron (BSE) and/or Cathodoluminescence (CL) imagery (Supplementary Figures 9 and 10).

Eight samples were analyzed in total, with up to six grains of each. Three samples (one with two age populations) produced robust age constraints (Supplementary Fig. 11). Smaller spot sizes were utilized to measure thin bright rims on one sample (LA4; 152, Supplementary Figs. 9 and 11), but the data lacked any measurable radiogenic Pb, and counts were mostly below detection. Five other samples were unsuccessful due to a lack of radiogenic lead (Supplementary Fig. 12).

Quality control was provided by repeat measurements of a carbonate material that has been adopted as a secondary reference material by multiple labs and has existing precise age determinations. Duff Brown Tank, here labelled DuffBrown, has an age

of 64.04 ± 0.67 Ma (2s, n=6; Hill et al., 2016). Pooled measurements from all sessions here provide an unanchored age of 62.30 ± 0.78 Ma (n=62; Supplementary Figure 13), and when anchored to the measured initial $^{207}Pb/^{206}Pb$ composition of Hill et al. (2016) produce an age of 66.76 ± 0.72 Ma (2s, analytical uncertainty only).

Supplementary Figures

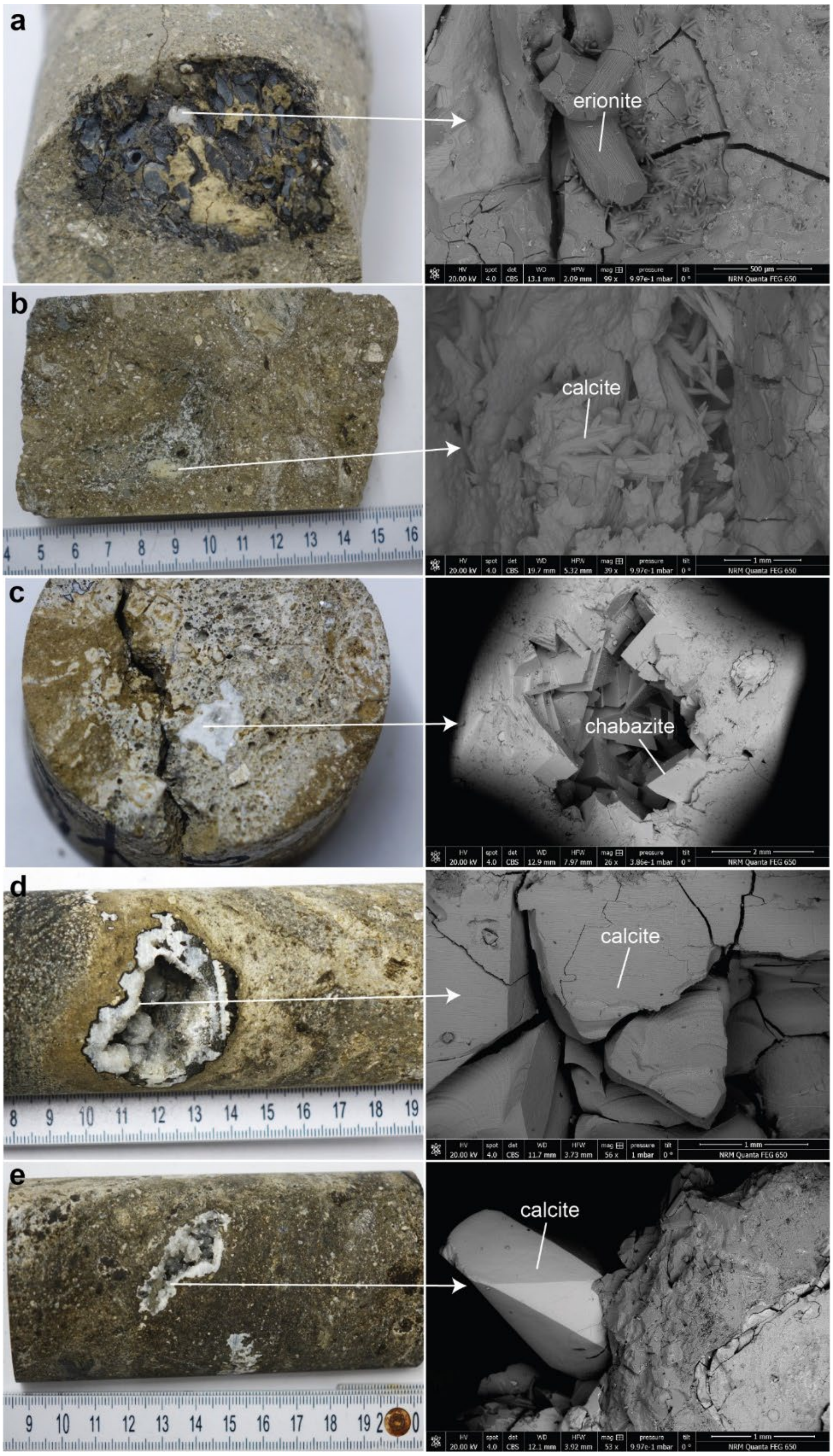

Supplementary Figure 1. Impact melt-bearing breccia samples with mineral-filled vugs within impact glass of various degrees of alteration. Mineral details in BSE-SEM photos. **a)** Sample LA4;26: **b)** Sample LA4;50 **c)** Sample LA4;59 **d)** Sample LA4;142 **e)** Sample LA4;151.

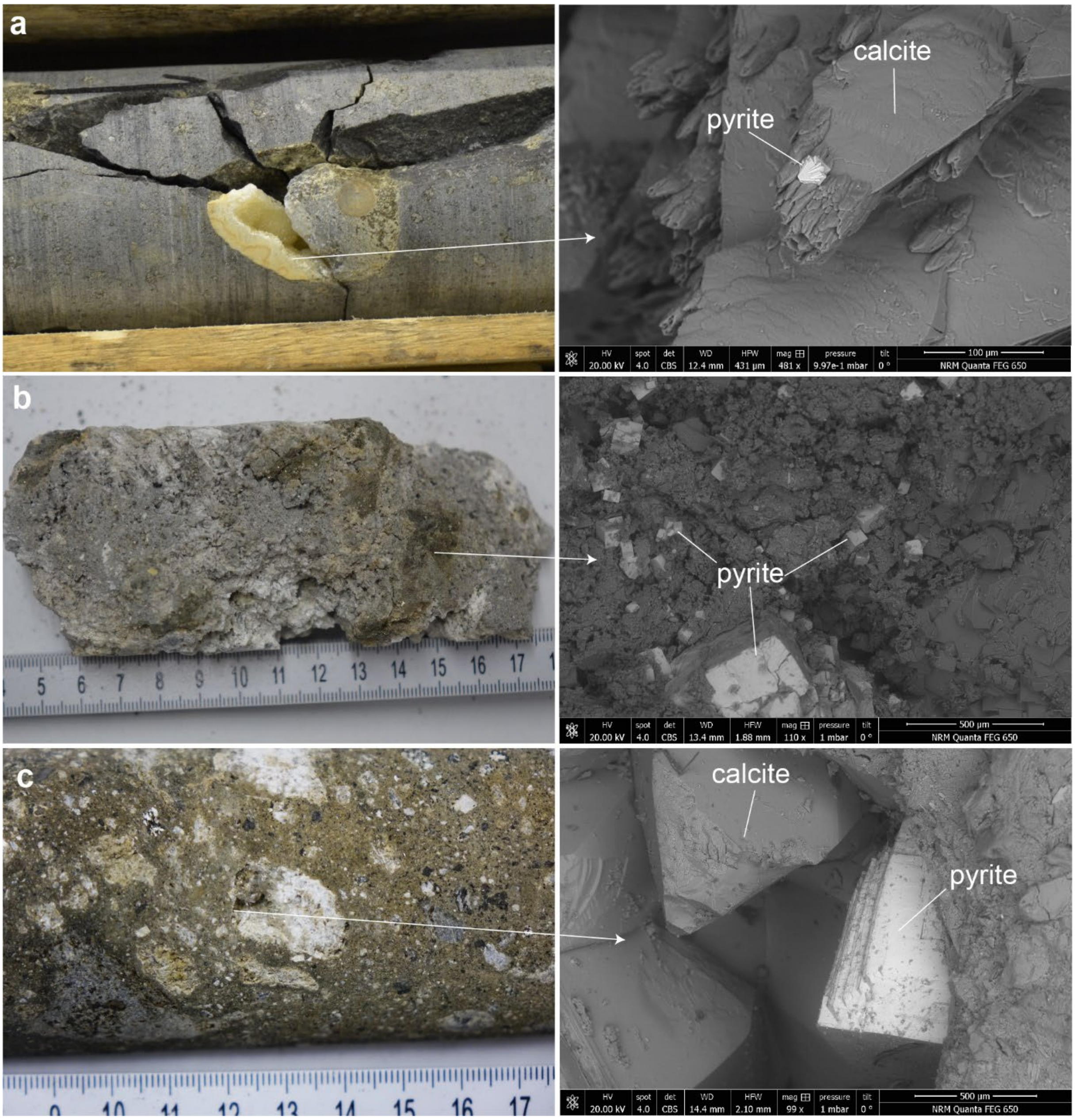

Supplementary Figure 2. Petrographic relationships of calcite and pyrite. Mineral details in BSE-SEM photos. **a)** Sample LA1;4: Pyrite intergrown with a calcite crystal. **b)** Sample LA4;130: Cubic pyrite in lithic impact breccia. **c)** La4;152: Cubic pyrite adjacent to scalenohedral calcite crystals in a vug of the impact melt-bearing breccia.

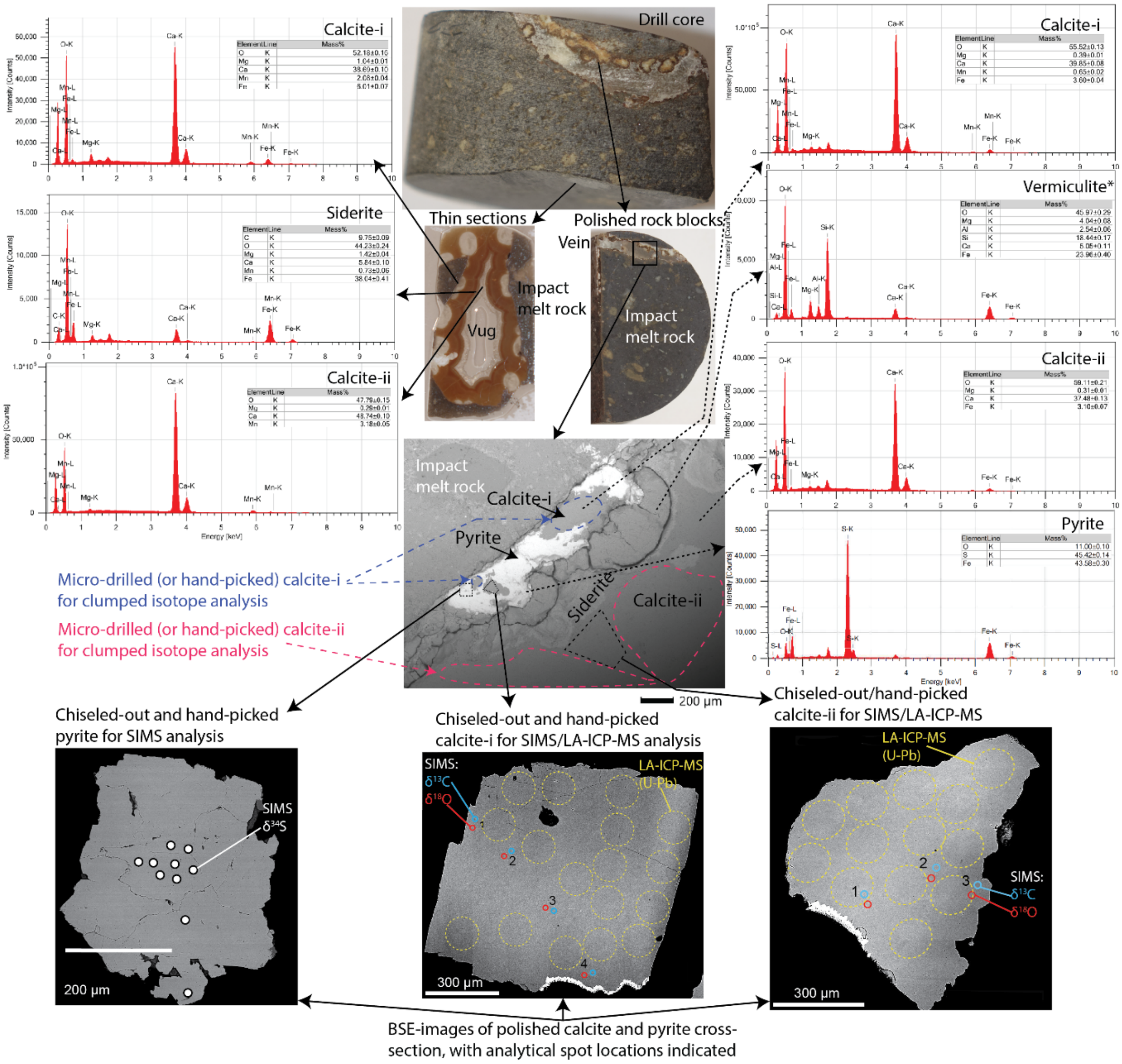

Supplementary Figure 3. Mineralogical details of paragenesis, and analytical scheme/relation for the mineral materials. For veins/vugs in the impact melt rock, here from La1:137, thin sections and rock blocks were prepared. Petrographic observations and grouping into generations were done by SEM-EDS observations and spectra. Minerals were hand-picked/chiseled, mounted in epoxy, polished to obtain cross-sections that were examined in SEM-EDS, followed by spot analysis with SIMS ($\delta^{34}S_{pyrite}$, $\delta^{18}O_{calcite}$, $\delta^{13}C_{calcite}$), followed by LA-ICP-MS spot analyses for U-Pb carbonate geochronology. For clumped isotopes, a corresponding part of the calcite filling/vug precipitate was hand-picked or micro-drilled for bulk analysis (mg sample volumes).

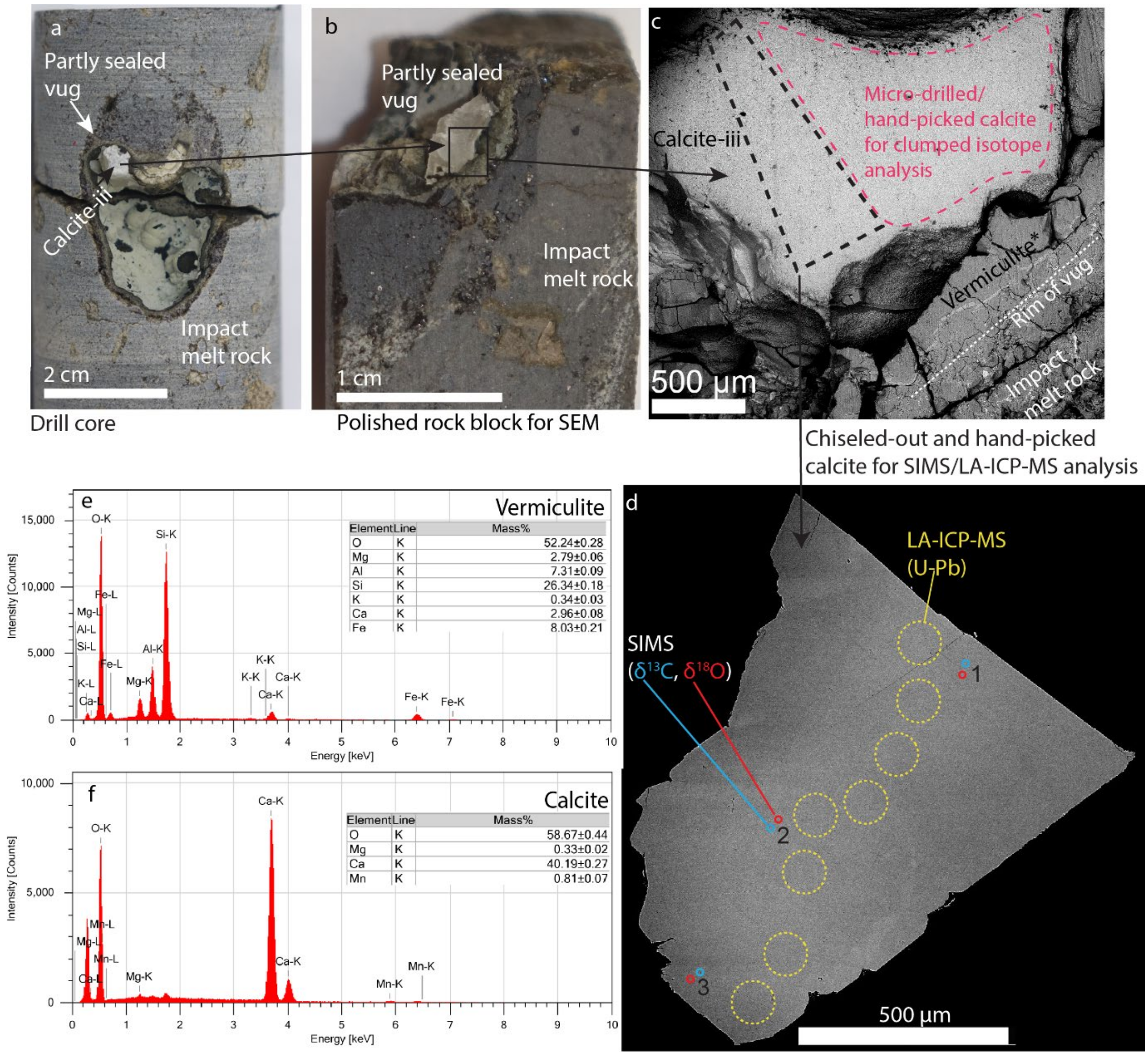

Supplementary Figure 4. Mineralogical details of paragenesis, and analytical scheme/relation for the mineral materials. For vugs (**a**) in the impact melt rock holding calcite-iii (sample La1:30), thin sections [not shown] and rock blocks were prepared (**b**). Petrographic observations and grouping into generations were done by SEM-EDS (**c**) observations and spectra (**e, f**). Minerals were hand-picked/chiseled, mounted in epoxy, polished to obtain cross-sections that were examined in SEM-EDS (**d**), followed by spot analysis with SIMS ($\delta^{34}S_{pyrite}$ [not shown], $\delta^{18}O_{calcite}$, $\delta^{13}C_{calcite}$), followed by LA-ICP-MS spot analyses for U-Pb carbonate geochronology. For clumped isotopes, a corresponding part of the calcite filling/vug precipitate was hand-picked or micro-drilled for bulk analysis (mg sample volumes).

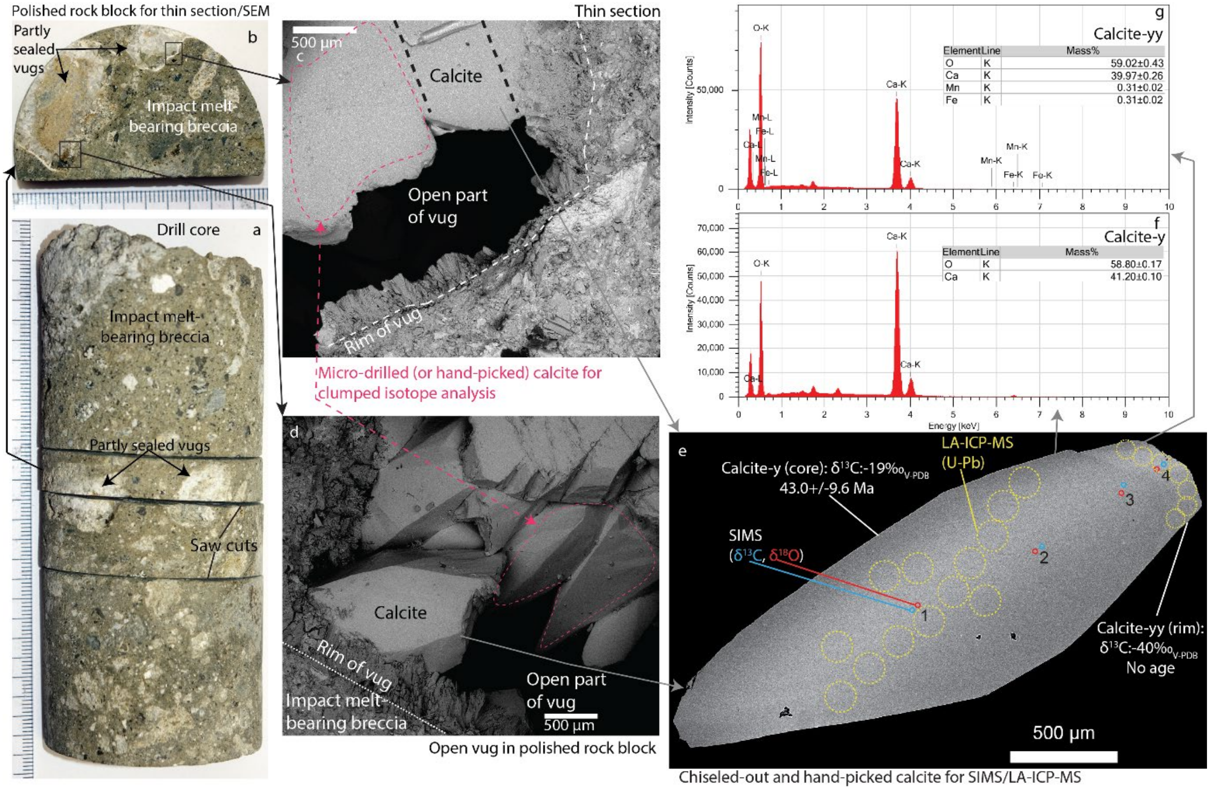

Supplementary Figure 5. Mineralogical details of paragenesis, and analytical scheme/relation for the mineral materials. For partly sealed vugs in the impact melt breccia (**a**), here from La4:152 (holding calcite-y and calcite-yy), thin sections and rock blocks (**b)** were prepared. Petrographic observations and grouping into generations were done by SEM-EDS observations (**c, d**) and spectra (**f, g**). Minerals were hand-picked/chiseled, mounted in epoxy, polished to obtain cross-sections that were examined in SEM-EDS (**e**), followed by spot analysis with SIMS ($\delta^{34}S_{pyrite}$ [not shown], $\delta^{18}O_{calcite}$, $\delta^{13}C_{calcite}$), followed by LA-ICP-MS spot analyses for U-Pb carbonate geochronology. For clumped isotopes, a corresponding part of the calcite filling/vug precipitate was hand-picked or micro-drilled for bulk analysis (mg sample volumes).

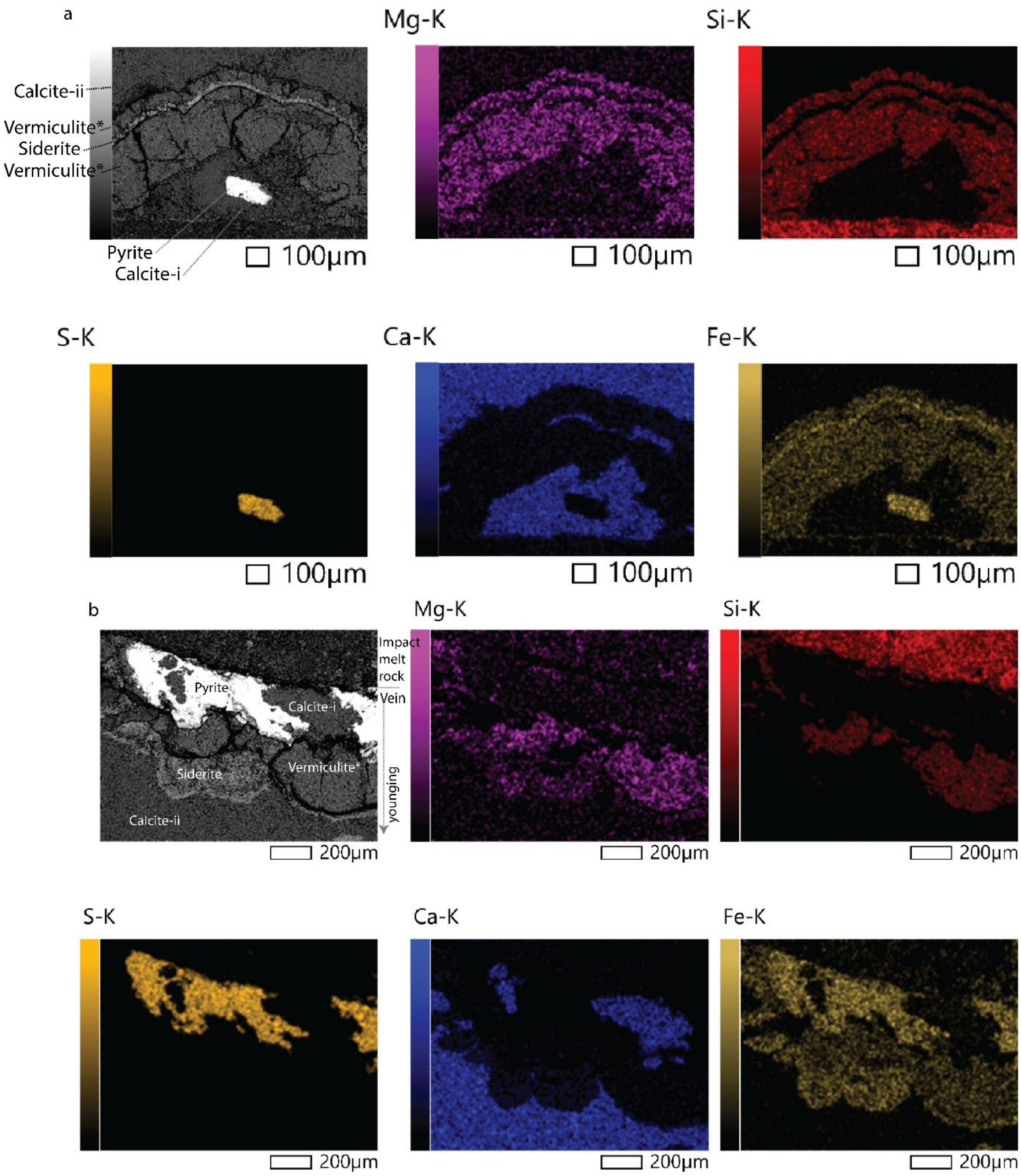

Supplementary Figure 6. EDS-maps of two sections from LA1:137 showing the petrographic relation of the vein minerals, such as pyrite embedded in calcite-i, **a)** and vice versa **b)** as well as the precipitation order: calcite-i/pyrite, vermiculite*, siderite (in mixed with vermiculite*, and/or later), and calcite-ii.

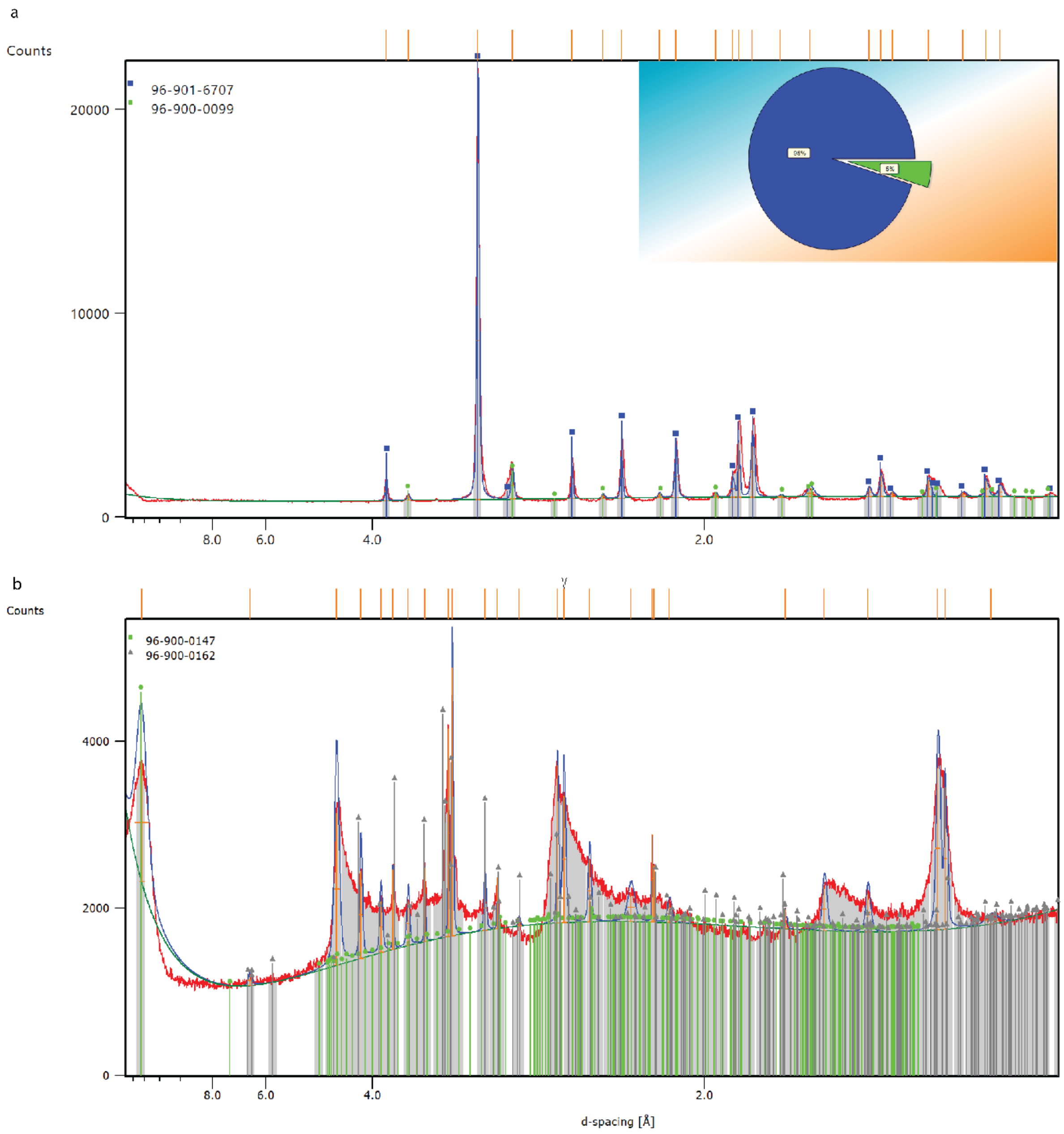

Supplementary Figure 7. X-ray diffractogram of **a**) a mixed sample of clearly defined peaks for siderite (COD: 96-900-0099, Graf, 1961) and calcite (COD: 96-901-6707, Ondrus et al., 2003) (sample LA1: 137) and **b)** clay mineral coating in sample LA1:30, showing peaks for poorly crystalline phase which is best fitted to vermiculite (COD: 96-900-0147,Shirozu and Bailey 1966) and orthoclase (COD: 96-900-0162, Colville and Ribbe, 1968). Peaks are listed in Supplementary Data 8.

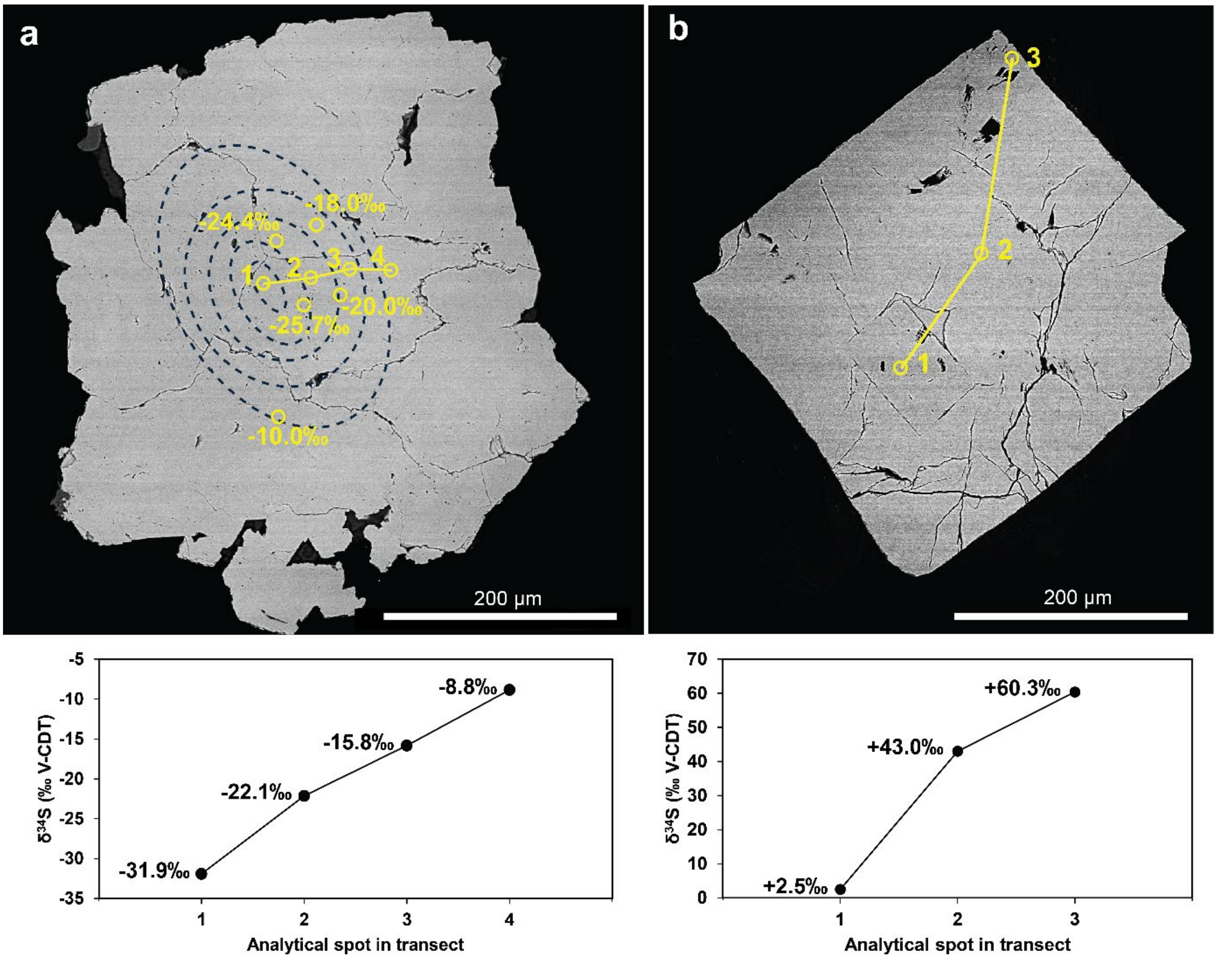

Supplementary Figure 8. S-isotope transects from core to rim of pyrite crystals. The error of $\delta^{34}S$ (1 σ) is within the size of the symbols. Each value represents one measurement. **a)** A ^{34}S-depleted pyrite crystal from the impact melt rock sample LA1;137, from pyrite intergrown with the first generation of calcite in this sample (i.e. with calcite group i in Fig. 2a of the main text). The diagram for the latter transect shows increased $\delta^{34}S$ values with growth. **b)** A ^{34}S-enriched pyrite crystal from the lithic impact breccia sample LA4;130 (petrographic context shown in Supplementary Fig 2b). The diagram for the latter transect shows increased $\delta^{34}S$ values from crystal core to rim.

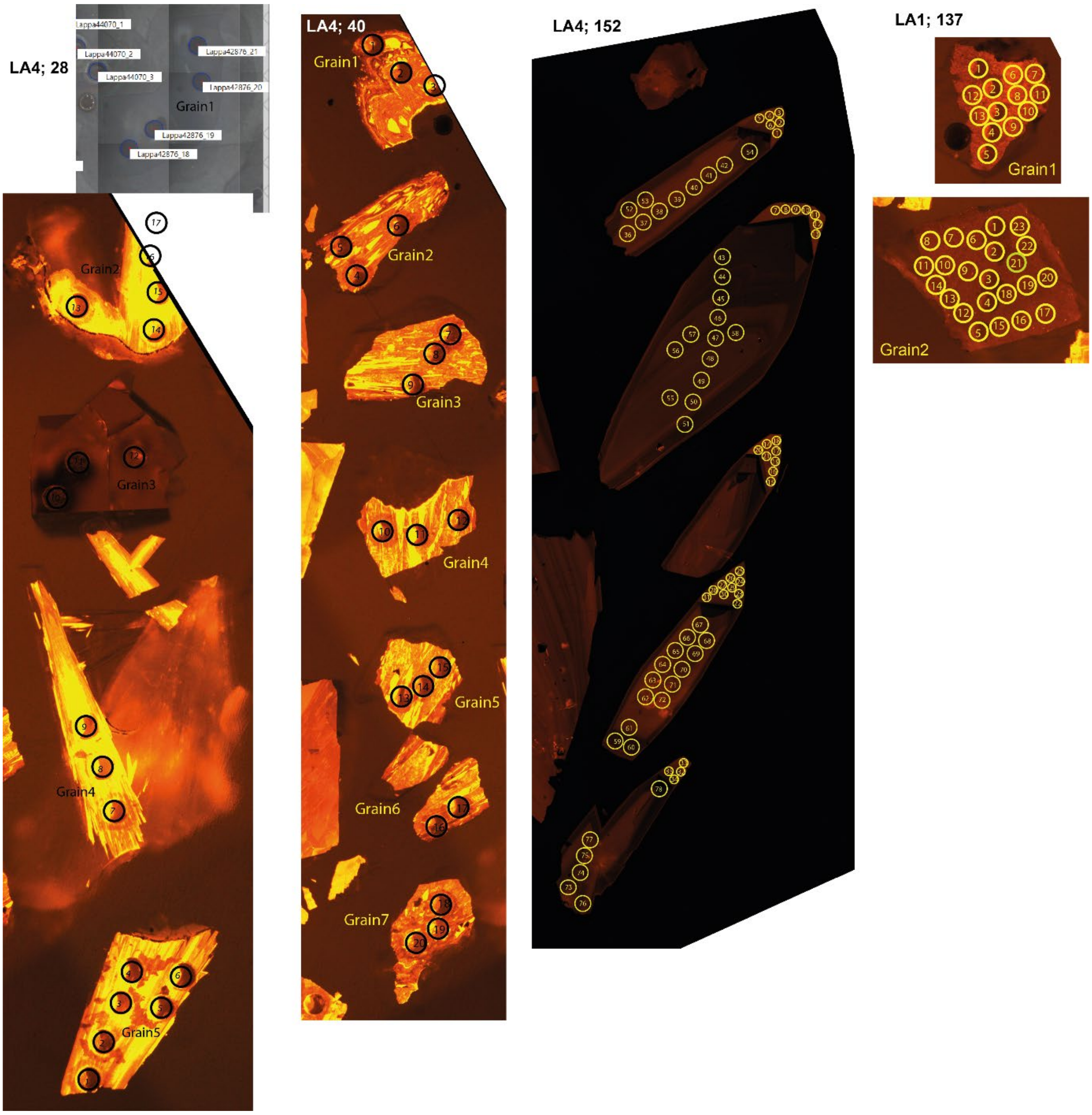

Supplementary Figure 9. CL images and U-Pb spot locations of analyzed calcite samples. All spots are 120 µm in diameter, except the smaller spots on LA4;152 rims, which are 60 µm in diameter. Analytical data are reported in Supplementary Data 6.

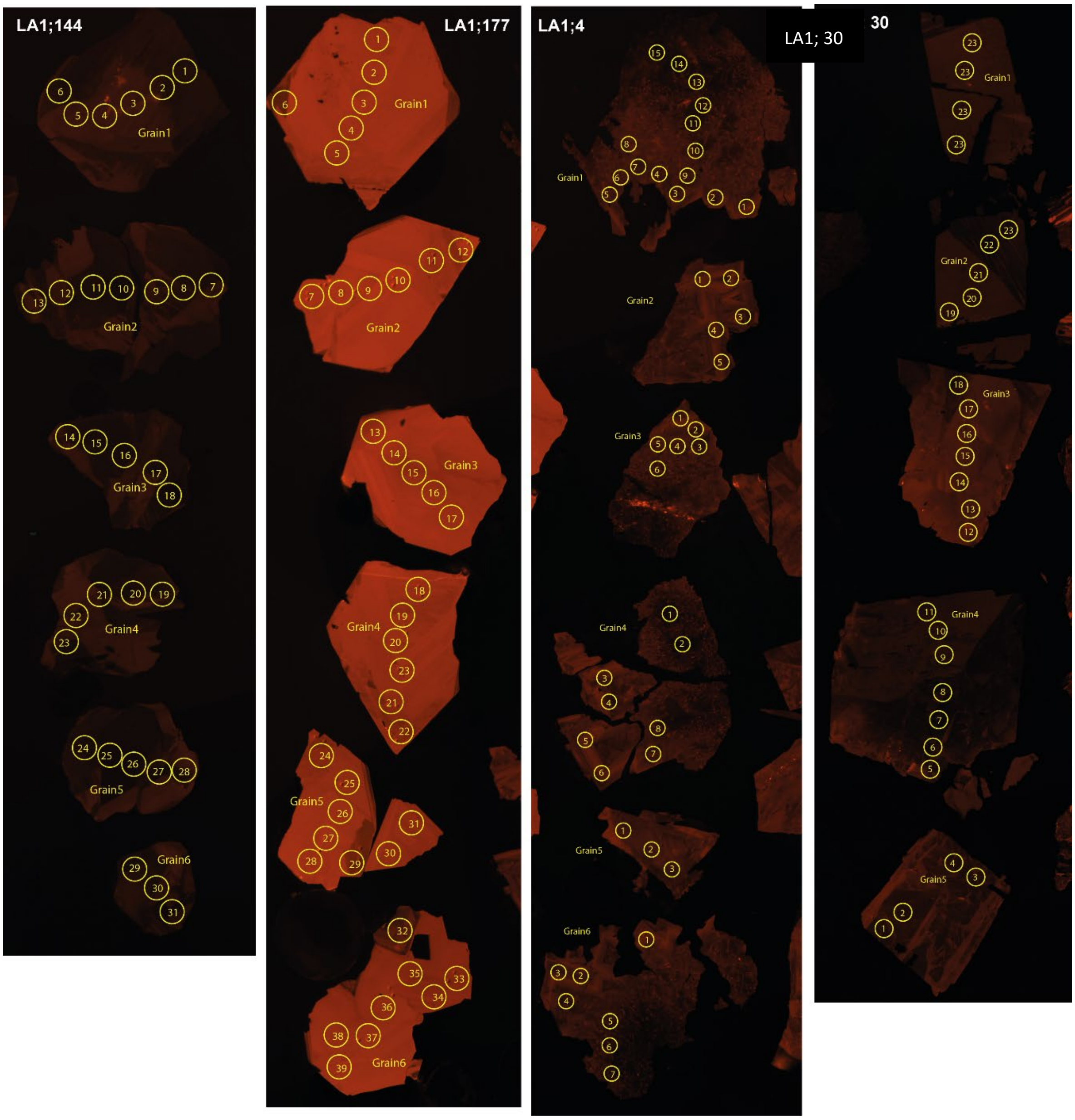

Supplementary Figure 10. CL images and U-Pb spot locations of analyzed calcite samples. All spots are 120 μm in diameter. Analytical data are reported in Supplementary Data 6.

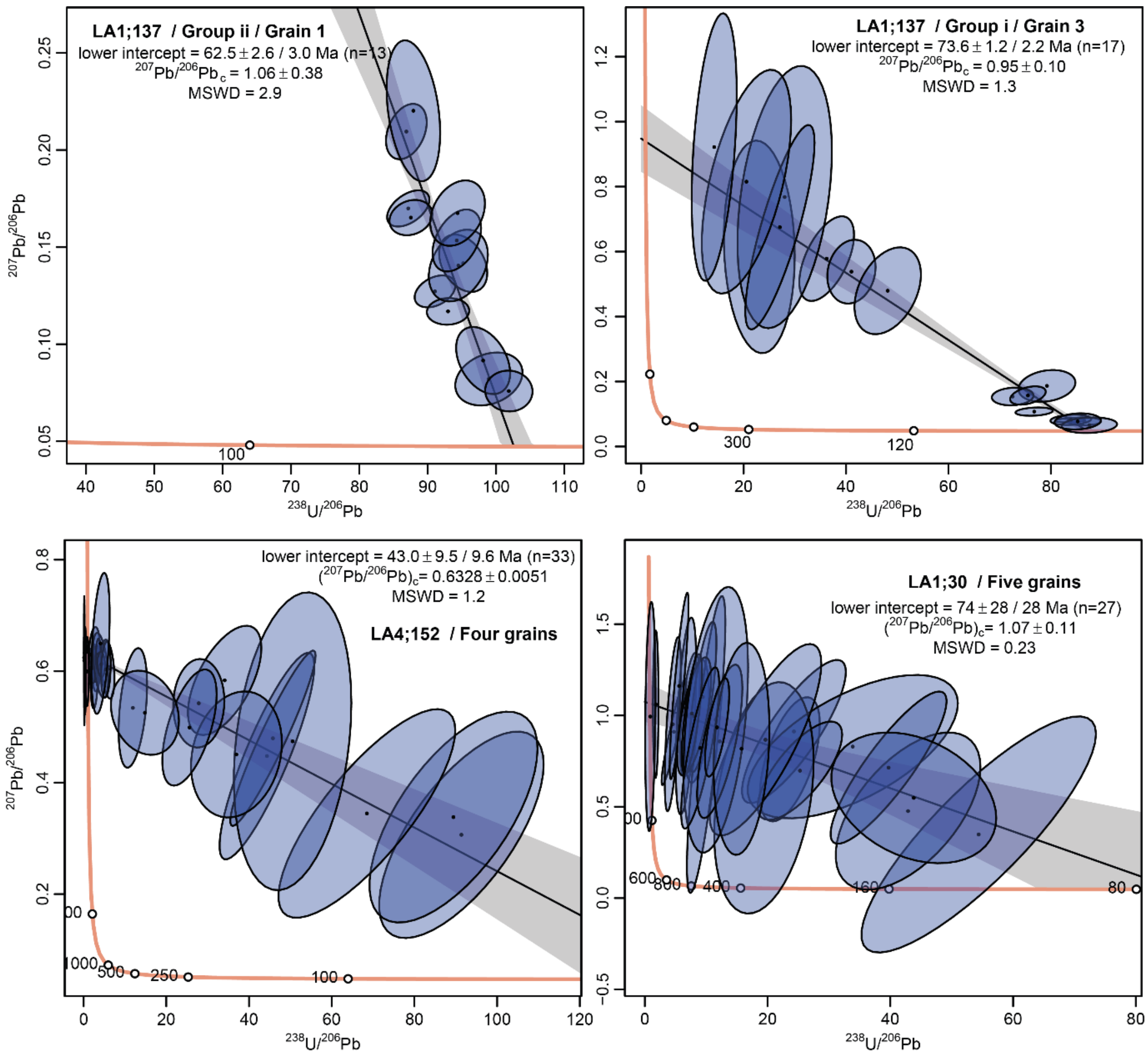

Supplementary Figure 11. Tera-Wasserburg U-Pb plots for successful samples; ellipses and uncertainties are 2σ. Age uncertainties are quoted as x / y, where x = analytical uncertainties, and y = analytical + systematic uncertainties. Analytical data are reported in Supplementary Data 6.

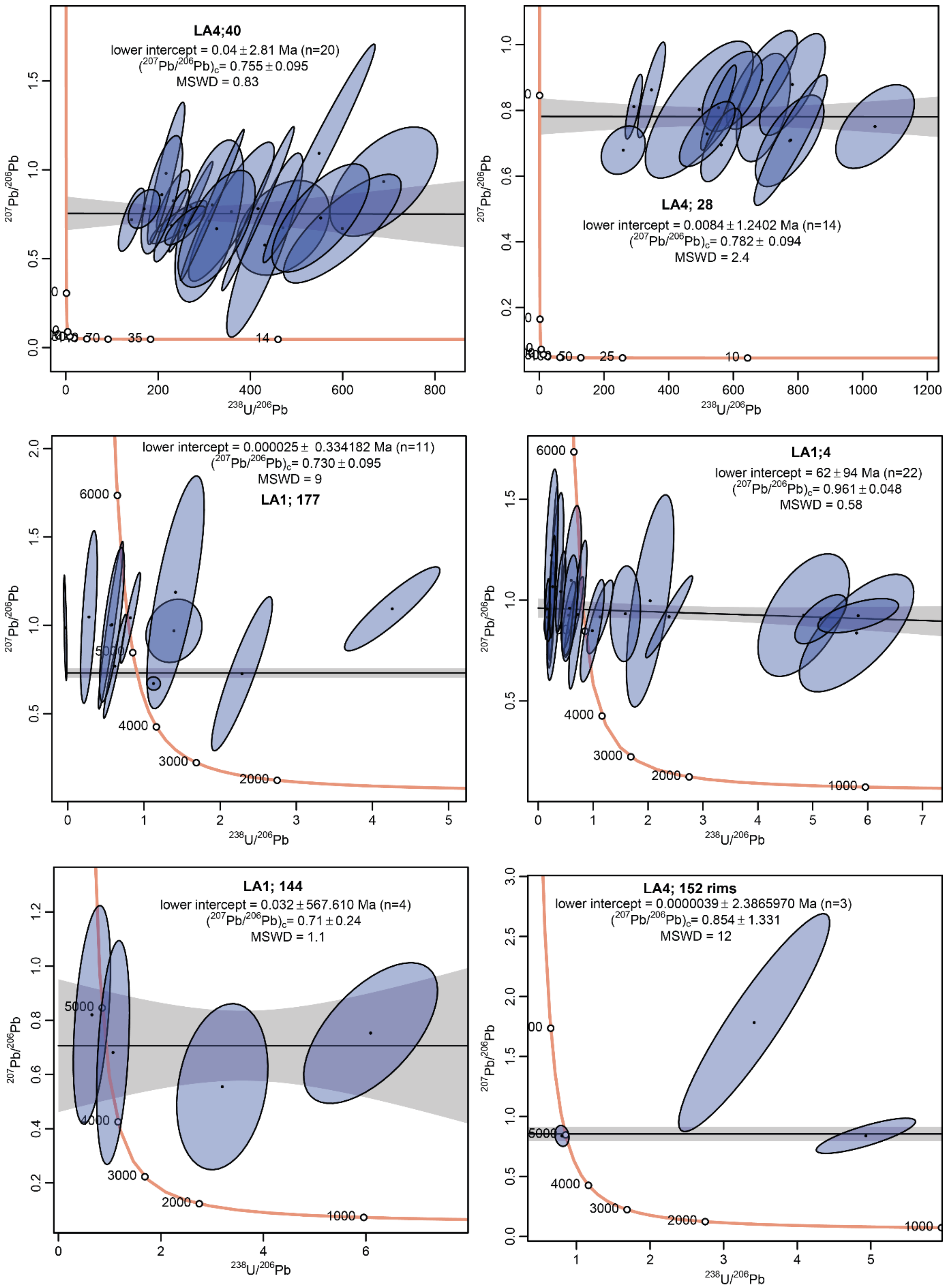

Supplementary Figure 12. Tera-Wasserburg U-Pb plots for unsuccessful samples; ellipses and uncertainties are 2σ. Age uncertainties are quoted as analytical uncertainties only. Analytical data are reported in Supplementary Data 6.

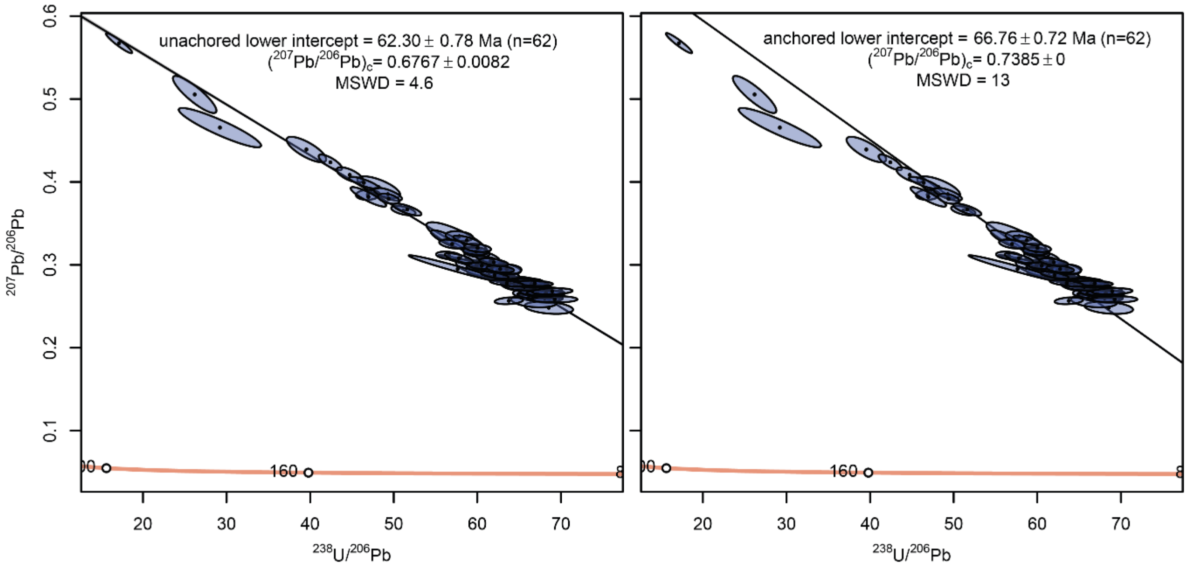

Supplementary Figure 13. Results of the secondary reference material DuffBrown from all sessions, plotted as Tera-Wasserburg U-Pb plots, and without (left) and with (right) anchoring to a defined common lead composition (from Hill et al., 2016).

References to the Supplementary information

1. Abramov, O. & Kring, D. A. (2004). Numerical modeling of an impact-induced hydrothermal system at the Sudbury crater. Journal of Geophysical Research. E. Planets 109, E10007.10001-n/a. https://doi.org/10.1029/2003JE002213
2. Abramov, O. & Kring, D. A. (2007). Numerical modeling of impact-induced hydrothermal activity at the Chicxulub crater. Meteoritics & planetary science 42, 93-112. https://doi.org/10.1111/j.1945-5100.2007.tb00220.x
3. Arp G., Kolepka C., Simon K., Karius V., Nolte N., and Hansen B. T. (2013). New evidence for persistent impact generated hydrothermal activity in the Miocene Ries impact structure, Germany. Meteoritics & Planetary Science 48:2491–2516.
4. Colville, A. A., Ribbe, P. H. (1968). The crystal structure of an adularia and a refinement of the structure of orthoclase, American Mineralogist, 53, 25 – 37.
5. Drake, H.; Heim, C.; Roberts, N.M.W.; Zack, T.; Tillberg, M.; Broman, C.; Ivarsson, M.; Whitehouse, M.J.; Åström, M.E. (2017). Isotopic evidence for microbial production and consumption of methane in the upper continental crust throughout the Phanerozoic eon. Earth Planet. Sci. Lett. 470, 108–118.
6. Graf, D. L., (1961) Crystallographic tables for the rhombohedral carbonates, American Mineralogist, 46, 1283 – 1316.
7. Graup, G. (1999). Carbonate–silicate liquid immiscibility upon impact melting: Ries Crater, Germany: Meteoritics & Planetary Science, v. 34, p. 425–438.
8. Gražulis, S., Chateigner, D., Downs, R. T., Yokochi, A. F. T., Quirós, M., Lutterotti, L., Manakova, E., Butkus, J., Moeck P., Le Bail, A. (2009). Crystallography Open Database–an open-access collection of crystal structures. Journal of applied crystallography, 42(4), 726-729.
9. Grieve, R.A.F., Dence, M.R., Robertson, P.B. (1977). Cratering processes: As interpreted from the occurrences of impact melts, in: Roddy, D.J., Pepin, R.O., Merrill, R.B. (Eds.), Impact and Explosion Cratering. Pergamon Press, New York, pp. 791–814.

10. Hall, A. M., Putkinen, N., Hietala, S., Lindsberg, E., & Holma, M. (2021). Ultra-slow cratonic denudation in Finland since 1.5 Ga indicated by tiered unconformities and impact structures. Precambrian Research, 352, 106000.
11. Hill, C. A., Polyak, V. J., Asmerom, Y., & P. Provencio, P. (2016). Constraints on a Late Cretaceous uplift, denudation, and incision of the Grand Canyon region, southwestern Colorado Plateau, USA, from U-Pb dating of lacustrine limestone. Tectonics, 35(4), 896-906.
12. Jõeleht, A., Kirsimäe, K., Plado, J., Versh, E. & Ivanov, B. (2005). Cooling of the Kärdla impact crater: II. Impact and geothermal modeling. Meteoritics & planetary science 40, 21-33. https://doi.org/10.1111/j.1945-5100.2005.tb00362.x
13. Kenny, G. G. et al. (2019). A new U-Pb age for shock-recrystallised zircon from the Lappajärvi impact crater, Finland, and implications for the accurate dating of impact events. Geochimica et Cosmochimica Acta 245, 479-494. https://doi.org/10.1016/j.gca.2018.11.021
14. Kenkmann, T. (2021). The terrestrial impact crater record: A statistical analysis of morphologies, structures, ages, lithologies, and more. Meteoritics & planetary science 56: 1024-1070. https://doi.org/10.1111/maps.13657
15. Kirsimäe, K., & Osinski, G. R. (2013). Impact-induced hydrothermal activity. Impact Cratering: Processes and Products, first ed. Wiley-Blackwell, New Jersey, 76-89.
16. Kukkonen, I., Kivekäs, L. & Paananen, M. (1992). Physical properties of kärnäite (impact melt), suevite and impact breccia in the Lappajärvi meteorite crater, Finland. Tectonophysics 216, 111-122. https://doi.org/10.1016/0040-1951(92)90159-4
17. Lehtinen, M. (1976). Lake Lappajärvi, a Meteorite Impact Site in Western Finland.
18. Leppäharju, N. (2008). Kalliolämmön hyödyntämiseen vaikut-tavat geofysikaaliset ja geologiset tekijät. Master's thesis,University of Oulu. 79 p. (in Finnish).
19. Mastrocicco, M.; Busico, G.; Colombani, N. (2018). Groundwater Temperature Trend as a Proxy for Climate Variability. Proceedings, 2, 630.
20. Merdith, A. S., Williams, S. E., Collins, A. S., Tetley, M. G., Mulder, J. A., Blades, M. L., ... & Müller, R. D. (2021). Extending full-plate tectonic models into deep time: Linking the Neoproterozoic and the Phanerozoic. Earth-Science Reviews, 214, 103477.
21. Naumov, M. V. (2005). Principal features of impact-generated hydrothermal circulation systems: Mineralogical and geochemical evidence. Geofluids, 5(3), 165-184.
22. Ondrus, P., Veselovsky, F., Gabasova, A., Hlousek, J., Srein, V., Vavrnn, I., Skala, R., Sejkora, J., Drabek, M. (2003). Primary minerals of the Jáchymov ore district, Journal of the Czech Geological Society, 48, 19 – 147.
23. Osinski, G.R., and Spray, J.G. (2001). Impact-generated carbonate melts: Evidence from the Haughton Structure, Canada: Earth and Planetary Science Letters, v. 194, p. 17–29.
24. Osinski, G. R., Lee, P., Parnell, J., Spray, J. G. & Baron, M. (2005). A case study of impact-induced hydrothermal activity: The Haughton impact structure, Devon Island, Canadian High Arctic. *Meteoritics & Planetary Science* 40, 1859-1877.
25. Osinski, G. R., Tornabene, L. L., Banerjee, N. R., Cockell, C. S., Flemming, R., Izawa, M. R., ... & Southam, G. (2013). Impact-generated hydrothermal systems on Earth and Mars. Icarus, 224(2), 347-363.
26. Osinski G. R., Grieve R. A. F., Ferrière L., Losiak A., Pickersgill A. E.**,** Cavosie A. J., Hibbard S. M., Hill P. J. A., Jaimes Bermudez J., Marion C. L., Newman J. D., and Simpson S. L**.** (2022). Impact Earth: A review of the terrestrial impact record. Earth-Science Reviews 232: 104112.
27. Reinhardt, M., Thiel, V., Duda, J. P., Hofmann, A., Bajnai, D., Goetz, W., ... & Drake, H. (2024). Aspects of the biological carbon cycle in a ca. 3.42-billion-year-old marine ecosystem. Precambrian Research, 402, 107289.

28. Roberts N. W. and Walker R. J. (2016). U-Pb geochronology of calcite mineralized faults; absolute dating of rift-related fault events on the northeast Atlantic margin. Geology 44, 531-534.
29. Roberts N. M. W., Rasbury E. T., Parrish R. R., Smith C. J., Horstwood M. S. A. and Condon D. J. (2017). A calcite reference material for LA-ICP-MS U-Pb geochronology. Geochemistry, Geophysics, Geosystems 18, 2807-2814.
30. Rochelle-Bates, N., Roberts, N. M. W., Sharp, I., Freitag, U., Verwer, K., Halton, A., ... & Schröder, S. (2021). Geochronology of volcanically associated hydrocarbon charge in the pre-salt carbonates of the Namibe Basin, Angola. Geology, 49(3), 335-340.
31. Rosberg, J. E., & Erlström, M. (2021). Evaluation of deep geothermal exploration drillings in the crystalline basement of the Fennoscandian Shield Border Zone in south Sweden. Geothermal Energy, 9, 1-25.
32. Royer, D. L., Berner, R. A., Montañez, I. P., Tabor, N. J., & Beerling, D. J. (2004). CO_2 as a primary driver of phanerozoic climate. GSA today, 14(3), 4-10.
33. Schmieder, M. & Jourdan, F. (2013). The Lappajärvi impact structure (Finland): Age, duration of crater cooling, and implications for early life. Geochimica et Cosmochimica Acta 112, 321-339. https://doi.org/10.1016/j.gca.2013.02.015
34. Sedighi, M., Bennett, D. P., Masum, S. A., Thomas, H. R., Johansson, E., & Siren, T. (2014). Analysis of temperature data at the Olkiluoto. (Posiva Working Report). Posiva Oy.
35. Shirozu, H., Bailey, S. W. (1966) Crystal structure of a two-layer Mg-vermiculite. American Mineralogist, 51, 1124 – 1143.
36. Timms, N.E., Erickson, T.M., Zanetti, M.R., Pearce, M.A., Cayron, C., Cavosie, A.J., Reddy, S.M., Wittmann, A., Carpenter, P.K. (2017). Cubic zirconia in >2370°C impact melt records Earth's hottest crust. Earth Planet. Sci. Lett. 477, 52–58. https://doi.org/10.1016/j.epsl.2017.08.012
37. Trowbridge, A. J., Marchi, S., Osinski, G. R. & Taron, J. M. (2024). Modeling of the Impact-Generated Hydrothermal System at the Haughton Impact Structure. *Journal of geophysical research. Planets* 129. https://doi.org/10.1029/2023JE008267
38. Vaarma, M. & Pipping, F. (1997). Pre-Quaternary rocks of the Alajärvi and Evijärvi map-sheet areas (in Finnish with English summary). Explanation to the maps of Pre-Quaternary rocks. Sheets 2313 and 2314. Geological map of Finland 1: 100 000. *Geol. Surv. Finland.*
