# Supplementary material for: Deep microbial colonization during impact-generated hydrothermal circulation at the Lappajärvi impact structure, Finland
Source: Nat Commun. 2025 Sep 17;16:8270. doi: 10.1038/s41467-025-63603-y (PMC12443983; doi:10.1038/s41467-025-63603-y)
Supplement: Supplementary file 3 — Description of Additional Supplementary Files [file 41467_2025_63603_MOESM3_ESM.pdf]

**Description of Additional Supplementary Files**

File Name: Supplementary Data 1
Description: SIMS analysis of reference pyrite material along with raw data of the samples

File Name: Supplementary Data 2
Description: SIMS analysis of reference calcite material along with raw data of the samples

File Name: Supplementary Data 3
Description: Carbonate clumped isotope analysis report - Summary

File Name: Supplementary Data 4
Description: Carbonate clumped isotope analysis report - Full

File Name: Supplementary Data 5
Description: Calculated $\delta^{18}O$-fluid values

File Name: Supplementary Data 6
Description: Carbonate U-Pb analytical data of reference materials and samples

File Name: Supplementary Data 7
Description: Carbonate U-Pb analytical conditions

File Name: Supplementary Data 8
Description: X-ray diffraction data
